# Age-dependent changes in the expression of regulatory cell surface ligands in activated human T-cells

**DOI:** 10.1186/1471-2172-14-45

**Published:** 2013-10-01

**Authors:** David H Canaday, Karen E Parker, Htin Aung, Hui Emily Chen, Dariana Nunez-Medina, Christopher J Burant

**Affiliations:** 1 Geriatric Research Center Clinical Center (GRECC), Louis Stokes Cleveland VA, 10701 East Blvd., 44106 Cleveland, OH, USA; 2 Division of Infectious Diseases and HIV Medicine, University Hospitals of Cleveland and Case Western Reserve University School of Medicine,10900 Euclid Ave, 44106 Cleveland, OH, USA; 3 Department of Hospital Medicine, Cleveland Clinic Foundation, Cleveland, OH, USA; 4 Case Western Reserve University Frances Payne Bolton School of Nursing, Cleveland, USA

**Keywords:** Immunosenescence, CD4, CD8, Adaptive immune system, Anergy, Co-receptor, PD-1, CTLA-4, ICOS, Tim-3

## Abstract

**Background:**

The immune system consists of multiple preformed and more specific adaptive immune responses, which are all subject to both positive and negative regulation. Programmed cell death protein 1 (PD-1) is a cell surface ligand implicated in the induction of anergy, Inducible T-cell Costimulator (ICOS) plays a stimulatory role in the development of both CD4+ and CD8+ T-cells, Cytotoxic T-Lymphocyte Antigen 4 (CTLA-4) plays a role in inhibitory regulation of T-cell activity, and T cell immunoglobulin and mucin protein 3 (Tim-3) has been described as a negative regulatory molecule in CD4+ helper type 1 cells and CD8+ cytotoxic type 1 cells. Each of these ligands is induced with T-cell activation allowing greater opportunity to have a regulatory role.

**Results:**

Flow cytometry was used to quantitate the expression of PD-1, ICOS, CTLA-4 and Tim-3 in human T-cells from geriatric and younger subjects both at baseline and after *in vitro* induction by mitogen. The magnitude of expression of the molecules increased significantly on activated blasts after mitogen stimulation compared to their baseline levels in resting cells. The increase in CTLA-4 expressing CD8+ T-cells was significantly higher after *in vitro* induction in older persons, while the increase in cells expressing Tim-3 and PD-1 was significantly reduced. In CD4+ T-cells, a greater increase in CTLA-4 expressing cells in older persons was the only difference between the age groups.

**Conclusions:**

We found several significant changes in the older individuals in regulatory elements of the adaptive immune system that occur particularly after immune activation. These differences could have ramifications to autoimmunity as well as immunology against infection and tumors.

## Background

Immuno-dysregulation in older adults leads to increased incidence of autoimmune diseases and decreased responses to vaccines such as influenza leading to higher vaccine failure rates [1,2]. If infected latently with *M. tuberculosis*, they are more likely to lose control of the infection and develop reactivation disease [3]. The positive and negative regulatory ligands and receptors may play a role in these changes.

PD-1 is a cell surface ligand expressed to some degree on resting and exhausted T-cells that increases following T-cell receptor activation [4]. PD-1 binds to its ligands (PDL1 and 2) to induce both T-cell apoptosis and anergy through inhibition of IL-2 production [5,6]. ICOS and CTLA-4 are both members of the CD28/B7 family of receptors [7]. ICOS plays a stimulatory role in the development of both CD4+ and CD8+ cells, and its deficiency leads to a decrease in numbers of memory T-cells. CTLA-4 plays a role in the inhibitory regulation of T-cell activity at least partially through its role in the suppressive functions of Tregs, and is thought to have a central role in the maintenance of peripheral tolerance [8].

The categorization of Tim-3 as either an inhibitory or stimulatory co-receptor is less straightforward. Tim-3 was first identified as a T-cell cell surface receptor involved in the regulation of Th1 responses, but has subsequently been shown to be expressed and active in antigen-presenting cells as well [9]. Tim-3 has been described as a negative regulatory molecule in CD4+ helper type 1 cells and CD8+ cytotoxic type 1 cells, possibly by inducing cell death in the cells expressing it or by promoting development of exhaustion [10]. Dysregulation of Tim-3 function has been implicated in the autoimmune disease multiple sclerosis [11].

It has been reported that the levels of inhibitory co-receptors in mouse T-cells are increased in aging animals [12,13], which could explain a general reduction in immunity and increase in susceptibility to autoimmune diseases in aging. To elucidate changes in the adaptive immune system that occur with aging related to regulatory molecules, we report here the levels of PD-1, CTLA-4, ICOS and Tim-3 in human peripheral T-cells in young versus older adults after mitogenic activation.

## Results and discussion

**Figure 1:**
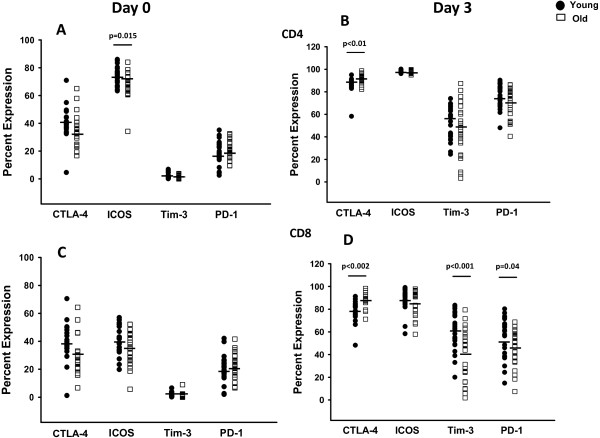
**Percent of cells expressing CTLA-4, ICOS, Tim-3 or PD-1 in young and older subjects.** CD4+ T-cells day 0 **(A)** and day 3 of activation **(B)**. CD8+ T-cells day 0 **(C)** and day 3 of activation **(D)**. Day 3 cells were gated on CD25+ blasts and day 0 on resting lymphocytes. Percentage positive determined by Overton method compared to isotype control. Mean indicated with the black bar. Each age group has n = 24 in ICOS, Tim-3, PD-1 and n = 19 for CTLA-4. Significant differences between young and older groups at the p ≤ 0.05 level (two-tailed t-test) are noted.

**Figure 2:**
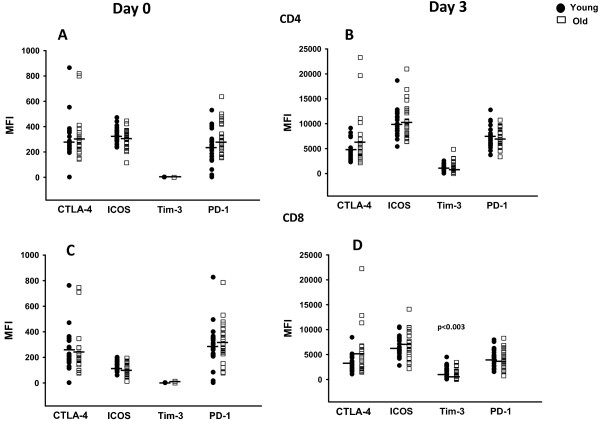
**MFI of PD-1, ICOS and CTLA-4 in young and older subjects.** CD4+ T-cells day 0 **(A)** and day 3 of activation **(B)**. CD8+ T-cells day 0 **(C)** and day 3 of activation **(D)**. Day 3 cells were gated on CD25+ blasts and day 0 on resting lymphocytes. MFI shown has isotype control MFI subtracted. Mean indicated with the black bar. Each age group has n = 24 in ICOS, Tim-3, PD-1 and n = 19 for CTLA-4. Significant differences between young and older groups at the p ≤ 0.05 level (two-tailed t-test) are noted.

We examined the proportion of CD4+ and CD8+ T-cells expressing the regulatory markers compared to isotype control and the mean fluorescence intensity (MFI) of the expression at day 0 and then after 3 days of activation. Figure 1A and C demonstrate that at day 0 baseline there were similar proportions of cells that expressed each one of these markers between the young and old except the percentage of ICOS expressing CD4+ T-cells were higher in young than old (74+ vs. 68%, p = 0.015). In both age groups and cell types after activation, the proportion of cells expressing each marker on activated blasts increased somewhat (Figure 1B and D) compared to baseline resting cells, but the MFI increase on blasts was more substantial (Figure 2B and D). In mitogen stimulated cells after 3 days, CD8+ T-cells (Figure 1D) had several significant differences between the young and old groups that included less Tim-3 expressing cells in the older subjects (60% vs. 39%, p < 0.001) and as well as a decrease in PD-1 expressing cells (55% vs. 45%, p = 0.04). On the other hand, CTLA-4 expressing cells (88% vs. 79%, p < 0.002) were increased in the older subjects compared to the younger ones. The MFI on the activated CD4+ and CD8+ T-cells was increased substantially for all molecules and in both age groups (Figure 2). The actual difference in MFI attained after activation with SEB however was only statistically different in the expression of Tim-3 on CD8+ T-cells with higher expression on the younger subjects (MFI 1860 vs. 889, p < 0.003). For these studies we gated on CD25+ blasts to compare the proportions of cells expressing the regulator molecules. This method was chosen to focus on the expression of the cells that become activated that can potentially effect a specific change in the immune response.

Our observations demonstrate that the most significant differences between T-cells in older and younger adults are in CD8+ cells. The differences demonstrated here in expression of Tim-3 and ICOS on T-cells between old and young after activation are novel. Similar to our study Leng et al. found significantly increased CTLA-4 after activation however our data found a similar increase in CTLA-4 regardless of age while their study had a greater increase in CTLA-4 with age [14]. This difference is possibly explained by a kinetic or technical difference involving the stimulation and antibody staining. Dolfi et al. recently reported that resting naïve CD8+ cells have increased PD-1 expression in older subjects, but they did not see a difference in PD-1 expression in old vs. young subjects after a 5-hour activation with influenza [15]. Our study extends this finding looking at PD-1 expression after a 3-day stimulation. When focusing on activated CD8+ T cells, old individuals have a small decrease in the percent of cells expressing PD-1 but not in the MFI of expression between young and old subjects. This suggests at least on a per cell basis that the PD-1 expression after activation is similar between the age groups.

Our results contrast with reported results in murine splenocytes. Channappanavar et al. found small but significant increase in PD-1, ICOS, and CTLA-4 in CD4+ T-cells in older inbred C57 mice [12]. Shimada et al. found only elevated PD-1 and not CLTA-4 on CD4+ T-cells in older C57 mice [13]. In contrast to our data they did not see increases in PD-1 with immune activation. The differences could be species or tissue vs. blood related. It does highlight the importance of confirming animal model data in humans.

We stimulated PBMC with mitogen to create a large population of activated cells that are readily identifiable by their CD25 expression to allow us to focus on activated T-cells. The findings in this study point to potential differences in patterns of ligand expression that can be studied in future vaccine or pathogen specific systems in an antigen specific manner. They may also have relevance in the area of anti-tumor immunity and autoimmunity. Anti-CTLA-4 monoclonal antibody is Food and Drug Administration approved for therapy against malignant melanoma. Higher induced expression in both CD4+ and CD8+ T-cells could result in diminished immunity in older persons. These differences could result in clinically significant changes in chronic conditions that include malignancy, autoimmunity and persistent infections. These data demonstrate that this area of aging research requires further investigation.

## Conclusions

We found several significant changes in the older individuals in regulatory elements of CD4+ and CD8+ T cells. After *in vitro* stimulation the proportion of activated CD8+ T cells from older individuals was significantly increased in CTLA-4 and decreased PD-1 and Tim-3. These differences could have ramifications to autoimmunity as well as immunology against infection and tumors.

## Methods

Peripheral blood mononuclear cells (PBMC) were isolated and cryopreserved from older adults (n = 27, mean age 86 years, ranging from 69 through 94 years) and younger adult controls (n = 37, mean age 34 years, ranging from 22 to 44 years) after obtaining informed consent using the protocols approved by the Institutional Review Boards of the Veterans Affairs Hospital in Cleveland and Case Western Reserve University [16]. Individuals on immunomodulatory medications (steroids, chemotherapy, cytokine inhibitors), with recent infection or hospitalization were excluded. The older group primarily consisted of patients from the Cleveland VA outpatient geriatric clinic and the younger subjects were staff and students at the Cleveland VA and Case Western Reserve University. After thawing, PBMC were cultured for 3 days with or without the mitogen Staphylococcal enterotoxin B (SEB) at 1 μg/ml. Cells were stained on days 0 and 3 with a viability dye (fixable LIVE/DEAD violet, Invitrogen), CD8-V500 (BD Biosciences), anti-CD3 APC/CY7, CD4-Alexa 700, CD25-PE/CY7, anti-PD-1-PerCP/CY5.5, ICOS-FITC, CTLA4-APC, and Tim-3-PE (Biolegend). Staining for CTLA-4 was performed using Cytofix/cytoperm reagents (BD Biosciences) according to the manufacture’s instructions while all other markers were stained only for surface expression. CTLA-4 is only transiently upregulated on the cell surface and intracellular staining methods must be employed to measure the true magnitude of activation-induced upregulation [17]. Data was acquired on a LSR-II flow cytometer and analyzed using FlowJo software (Tree Star).

Comparison was made between the resting non-activated cells at day 0 and the activated CD25+ T-cells blasts at day 3. CD3+ cells were gated by size and then live dead staining. Cells were then subtyped into CD8+ and CD4+ T-cells. They were analyzed in FlowJo using the Overton method to determine the percent positive cells above isotype control. The reported MFI of PD-1, ICOS, CTLA-4 and Tim-3 has the MFI of the isotype-control stained cells subtracted. Statistical analysis was performed with SPSS (IBM) using t-tests. Levene’s test for equality of variances was applied to each comparison and the appropriate t-test results chosen.

## Abbreviations

CTLA-4: Cytotoxic T-Lymphocyte Antigen 4; ICOS: Inducible T-cell costimulator; MFI: Mean fluorescence intensity; PD-1: Programmed cell death protein 1; Tim-2: T cell immunoglobulin and mucin protein 3.

## Competing interests

The authors have no competing interests to report.

## Authors’ contributions

DHC conceived of the project and directed the study design. HEC, DNM, and HA performed the experiments. CB performed the statistics. KEP and DHC performed the final analysis of the data and wrote the paper. All authors read and approved the final manuscript.
